# Smithing Processes Based on Hammer Scale Excavated from the Third- to Fourth-Century Ancient Iron-Making Sites of the Korean Peninsula

**DOI:** 10.3390/ma15124188

**Published:** 2022-06-13

**Authors:** Dayeon Jung, Heehong Kwon, Namchul Cho

**Affiliations:** Department of Cultural Heritage Conservation Science, Kongju National University, Gongju 32588, Korea; jdyeon@korea.kr (D.J.); entasis@korea.kr (H.K.)

**Keywords:** Jincheon Songduri site, hammer scales, flake hammer scale, spheroidal hammer scale, smithing process

## Abstract

The by-products of iron smelting and smithing include slag, flake hammer scale, and spheroidal hammer scale. The analysis of such iron-making by-products reveals critical information regarding the development of iron culture and the process characteristics. Using a metallographic microscope, SEM-EDS, and Raman micro-spectroscopy, we investigated the manufacturing process by examining the microstructure and determining the composition of the flake hammer scale and spheroidal hammer scale excavated from Korean Peninsula sites of iron manufacture during the Proto-Three Kingdoms Period, in the third and fourth centuries CE. Microstructure analysis confirmed that as the process progressed, the flake hammer scale’s thickness decreased owing to forging, which flattened the structure. Additionally, three layers were observed, with the surface layer identified as hematite (Fe_2_O_3_), the middle layer identified as magnetite (Fe_3_O_4_), and the inner layer identified as wüstite (FeO). The analysis of hammer scales revealed that the forging process to create iron bars required repeated working, following a refining process to remove impurities, confirming the division of labor in the smithing process. Correspondingly, the smithing process stages can be deduced from the structural shape and thickness of the hammer scale produced during the iron manufacturing process. Thus, the findings of this study are expected to be invaluable in furthering our understanding of the smithing process in detail, through future research on hammer scale.

## 1. Introduction

Owing to the susceptibility of iron to corrosion, ancient iron artifacts rarely retain their original shapes. As a result, deciphering the development of iron culture through excavated iron artifacts is quite laborious [1]. In most cases, iron-manufacturing sites contain remnants of iron manufacturing or by-products derived from individual processes; thus, the by-products of a particular shape can be scientifically analyzed to determine the ironwork’s technology level at the time [2].

Iron-making is a production system comprising a number of processes and techniques, ranging from mining to the manufacture of ironware [3]. Iron production begins with mining operations to extract high-quality raw materials, such as iron ores or iron sand. Smelting is a process that separates iron from iron ore by reducing it in its natural state. There are direct and indirect methods for smelting. Iron in the oxide state can be reduced at temperatures as low as 800 °C, which is lower than iron’s melting point of 1539 °C. Because iron ores contain gangue components, such as silica and alumina, successful smelting requires the separation of these impurities in the form of slag [4]. Thus, it is critical to maintain a temperature between 1150 °C and 1350 °C, at which sufficient carbon monoxide can be produced under reduction conditions. However, this task was exceptionally difficult during the early days of smelting [5]. For direct smelting, a temperature of 1539 °C is required to obtain pure iron, but achieving this temperature in ancient times with only charcoal fuel and natural ventilation proved difficult [6]. Thus, direct smelting, a method for obtaining iron by reducing ores at temperatures of less than 1200 °C, was developed [7]. The process of indirect smelting entails reducing iron at temperatures greater than 1200 °C in a furnace [8,9]. The raw material is completely melted to separate the iron from the slag, which typically results in glassy slag [8,10].

**Figure 1:**
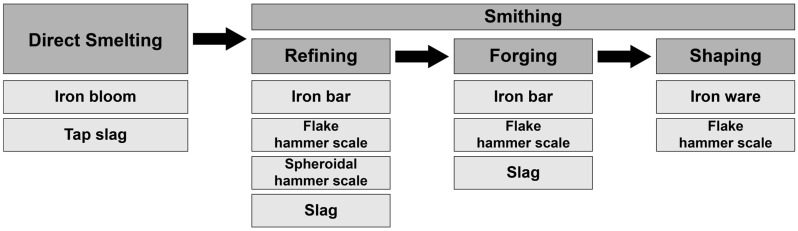
Direct smelting, as used in the iron-making process and iron by-products.

Smithing is a term used to describe the process of forging smelted iron bloom into ironware. It is classified as refining-smithing or forging-smithing, depending on the function and process of smithing (Figure 1). Direct-smelting iron blooms are not sufficiently reduced and contain a high concentration of impurities. Refining refers to the process of removing impurities from heated iron bars [4], while forging produces iron bars by repeated pounding, compressing impurities, and shaping the ironware to the desired shape. Smithing by-products include both spheroidal and flake hammer scales [11].

In Korea, research into the analysis of the flake hammer scale and spheroidal hammer scale that are generated during reproduction experiments has been ongoing. The following are some examples: a metallurgical study using reproduction experiments performing traditional smelting and smithing processes [12]; a characterization of individual processes using reproduction experiments [2]; a study of by-products using reproduction experiments performing refining and forge welding [13]; and a study on ancient iron-making technology using reproduction experiments [14,15]. Additionally, the hammer scale excavated from the Bokam-ri site in Naju [16], and the granular slags and hammer scale excavated from the Anti-Japanese Righteous Army’s historic site on Ssangsan Mountain in Hwasun [17] were analyzed for research into the iron-making material excavated from these historic sites. However, research on locations other than those mentioned previously is still lacking.

The characteristics of the smithing process were investigated in the current study, through a scientific analysis of the flake hammer scale and spheroidal hammer scale excavated from iron-making sites in Korea that were in production during the Proto-Three Kingdoms Period in the third and fourth centuries CE.

## 2. Site Overview

**Figure 2:**
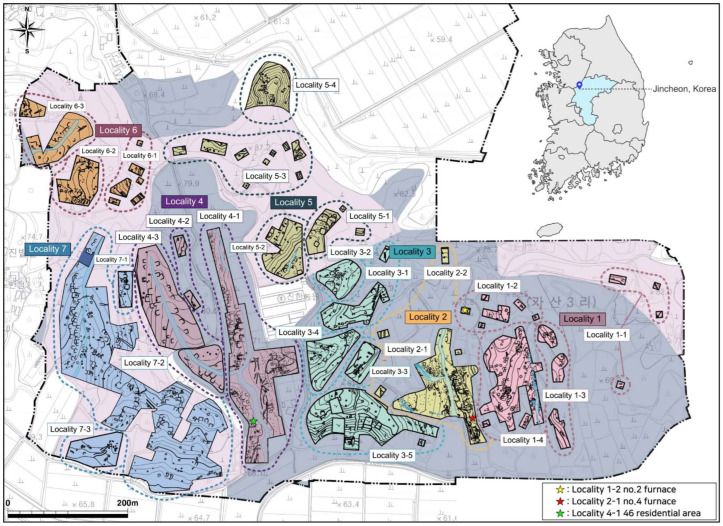
Arrangement of the remains found on the Jincheon Songduri site (Central Institute of Cultural Heritage, 2020).

A total of 56 remains from iron-making-related sites, including smelting and smithing furnaces, were investigated at the Songduri site between 2016 and 2018, via a sample survey and an excavation survey (Figure 2). The Proto-Three Kingdoms Period remains were primarily associated with daily life and production. The remains associated with daily life were primarily discovered around structures and residential areas, and included pits, moats, watershed facilities, and pillars, whereas the majority of the remains associated with iron production were earthenware kilns and iron-making-related remains [18]. Iron-making-related sites were discovered at points 1-2, 1-4, 2-1, 3-5, 4-1, 7-2, and 7-3, with the majority of them being concentrated along old waterways. Short-necked pots, bowl-shaped pottery, basins, iron sickles, iron axes, iron knives, and beads were discovered in Proto-Three Kingdoms Period residential areas. We divided the time period into three stages, based on the internal structures of residential areas, the shapes of the excavated pottery, comparison with nearby sites, and accelerator mass spectrometry (AMS) dating at the Jincheon Songduri site. Stage 1 corresponds to the early to mid-third century, Stage 2 to the mid- to late third century, and Stage 3 to the late third to early fourth centuries. The site was believed to have functioned only between the early third and fourth centuries, with the mid-third century period being the most significant [18]. The study examined flake hammer scales and spheroidal hammer scale excavated from the No. 2 furnace at Locality 1-2, flake hammer scale excavated from the No. 4 furnace at Locality 2-1, and residential area No. 46 at Locality 4-1.

## 3. Samples for Analysis and Analytical Methods

### 3.1. Samples for Analysis

**Figure 3:**
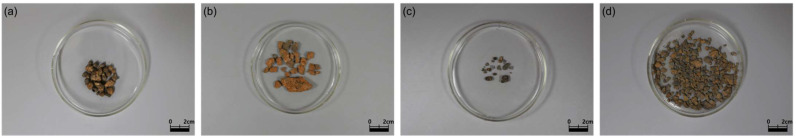
Flakes excavated from the Jincheon Songduri site: (**a**) spheroidal hammer scale, (**b**) flake hammer scale, (**c**) flake hammer scale, (**d**) flake hammer scale.

One piece of spheroidal hammer scale and three pieces of flake hammer scale were selected by archaeologists for analysis from the Jincheon Songduri site, dating from the Proto-Three Kingdoms Period, because they were closely related to the smithing process (Table 1, Figure 3). The spheroidal hammer scale sample No. 1 and the flake hammer scale sample No. 2 were both recovered from the same ironmaking-related remains.

### 3.2. Microstructure Analysis

Epoxy resins were used to mount the spheroidal hammer scale and flake hammer scale samples to observe their cross-sections. After polishing the mounted samples incrementally using mesh from 100 mesh to 4000 mesh, fine polishing was carried out using 3 µm and 1 µm DP-sprays (Struers, Copenhagen, Denmark) until no scratches were detected on the samples’ surface. A metallographic microscope was used to examine the microstructure of the polished samples (DM 2500M, Leica, Wetzlar, Germany). After examining the microstructure in detail with a scanning electron microscope (SEM, MIRA3, Tescan, Brno, Czech Republic), the chemical composition of the microstructure was determined using an energy-dispersive spectrometer (EDS, QUANTAX200, Bruker, Leipzig, Germany). EDS mapping was used to determine the distribution of elements in the microstructure of the spheroidal hammer scale (No. 1), which required in-depth analysis. To increase conductivity and minimize the effect on the composition ratio, all analyzed samples were coated with platinum (Pt).

### 3.3. Raman Micro-Spectroscopy

Raman micro-spectroscopy was used to accurately identify the microstructures (Lab Aramis, Ar-ion Laser 514.5 nm, Horiba Jobin Yvon, Palaiseau, France). Samples were prepared in advance for use in Raman micro-spectroscopy. Only the platinum (Pt) coating used in the previous microstructure analysis process was removed, and no additional pre-treatment was used to conduct the analysis.

## 4. Analysis Results

### 4.1. Spheroidal Hammer Scale (No. 1)

**Figure 4:**
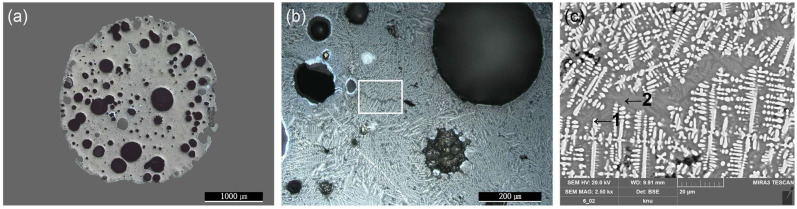
Microstructure and SEM image of spheroidal hammer scale (No. 1) (**a**) whole image, (**b**) metallographic microscope detailed image and rectangle is position of the SEM analysis, (**c**) position of SEM image and EDS analysis; 1: wüstite, 2: kirschsteinite.

**Figure 5:**
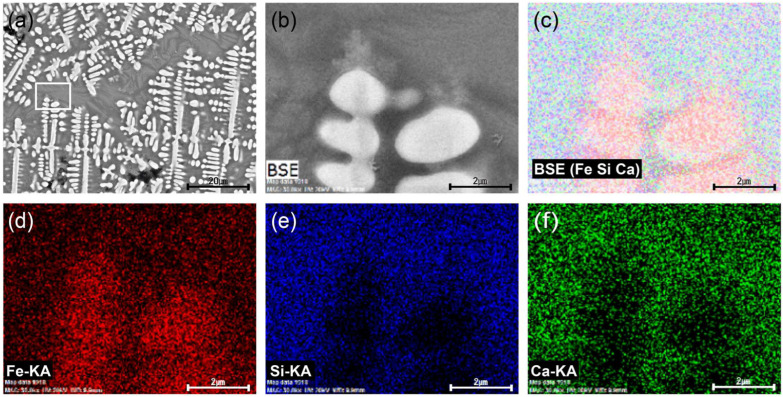
EDS mapping of spheroidal hammer scale (No. 1): (**a**) SEM image and position marking, (**b**) EDS mapping image, (**c**) distribution status of Fe, Ca, and Si elements, (**d**) Fe, (**e**) Si, (**f**) Ca.

**Figure 6:**
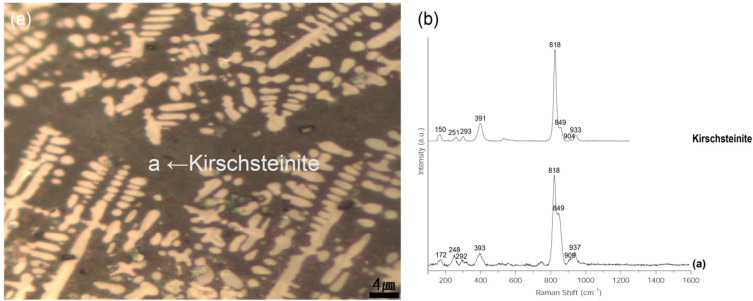
(**a**) Position of Raman micro-spectroscopy analysis of spheroidal hammer scale (No. 1), (**b**) Raman spectrum.

Spheroidal hammer scale (No. 1) is a type of iron material formed when ironware used in the smithing process is heated in a furnace and the surface materials, such as impurities, melt, flow down, and fall to form the slag [19]. There are numerous internal pores in the material, which is composed of a glassy matrix, dendritic wüstite, and columnar fayalite. The slag is separated during the refining–smithing process and cooled to form a flake hammer scale with a spherical shape due to surface tension [2]. Observation with a metallographic microscope (Figure 4a,b), confirmed the spherical shape, and large and small pores are scattered throughout the sample. Inside, a grey background and a densely developed white dendritic structure could be seen. SEM-EDS analysis (Figure 4c, Table 2) confirmed that the dendritic structure in EDS position 1 was made of wüstite, a mineral with a high Fe content. EDS position 2 contained a high concentration of Fe, Si, and Ca. The EDS mapping results shown in Figure 5 confirm that the Fe was concentrated in the white dendritic structure and that Fe, Si, and Ca were distributed throughout the grey background area. Raman micro-spectroscopy was used to precisely identify the microstructures (Figure 6). The 1-a structure with a grey background exhibited Raman shifts of 172, 248, 292, 393, 818, 849, 909, and 937 cm^−^^1^, which correspond to those of kirschsteinite (CaFeSiO_4_) [20].

### 4.2. Flake Hammer Scale (No. 2)

A flake hammer scale (No. 2) is a thin flake of approximately 1 mm in thickness that breaks away during the smithing process. It possesses strong magnetic properties and is used to evaluate smithing work, along with the spheroidal hammer scale [19]. The entirety of the flake hammer scale is composed of iron oxide particles, with trace amounts of fayalite or a glassy phase that formed along the iron oxide particle boundaries [21].

**Figure 7:**
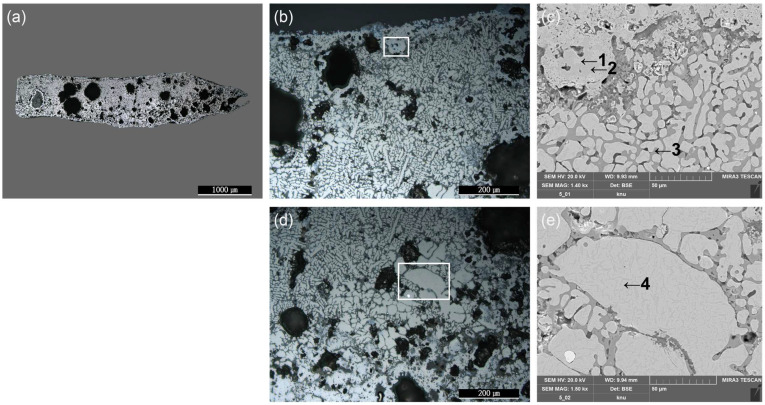
Microstructure and SEM image of flake hammer scale (No. 2): (**a**) whole image, (**b**,**d**) metallographic microscope detailed image and rectangle is position of the SEM analysis, (**c**,**e**) position of the SEM image and EDS analysis; 1: wüstite, 2: magnetite, 3: fayalite, 4: wüstite.

The metallographic microscope observations (Figure 7a,b,d) confirmed the presence of large pores on the inside of the flake, as well as the growth of white dendritic and granular structures on a grey background. The SEM-EDS analysis results (Figure 7c,e, Table 3) revealed that EDS positions 1, 2, and 4 had similar compositions, whereas position 3, which is the grey background, had elevated Fe, Si, and Ca contents.

**Figure 8:**
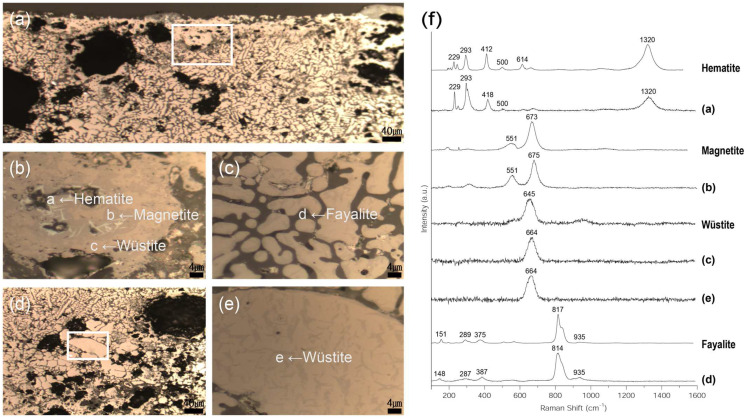
(**a**–**e**) Position of Raman micro-spectroscopy analysis of flake hammer scale (No. 2); (**a**) rectangle is position of (**b**,**c**), (**d**) rectangle is position of (**e**,**f**) Raman spectrum.

Raman microscopy was used to precisely identify the microstructures of similar components (Figure 8), and it enabled the determination of the difference in brightness shown in Figure 8b, which was not detectable using the metallographic microscope. Raman spectroscopy revealed Raman shifts of 297, 293, 418, 500, and 1320 cm^−1^ in structure 2-a, which was confirmed to be hematite (Fe_2_O_3_) [22]. Raman shifts of 551 and 675 cm^−1^ were also detected in structure 2-b, which was confirmed to be magnetite (Fe_3_O_4_) [23]. Furthermore, Raman micro-spectroscopy revealed a Raman shift of 664 cm^−1^ in structures 2-c and 2-e, which was confirmed to be wüstite (FeO) [24]. Additionally, Raman shifts of 148, 287, 387, 814, and 935 cm^−1^ were detected in the grey background, indicating that structure 2-d is of fayalite (Fe_2_SiO_4_) [25]. On the surface, hematite, magnetite, and wüstite were identified. Additionally, magnetite was discovered to coexist with wüstite on the inside of the sample.

### 4.3. Flake Hammer Scale (No. 3)

**Figure 9:**
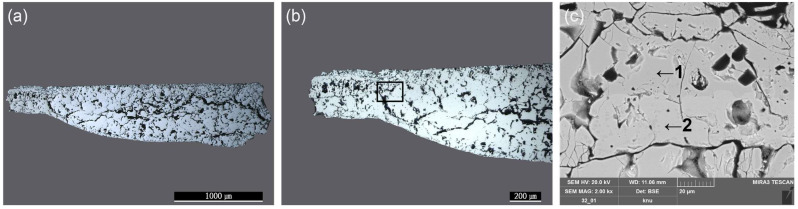
Microstructure and SEM image of flake hammer scale (No. 3): (**a**) whole image, (**b**) metallographic microscope detailed image and rectangle is position of the SEM analysis, (**c**) position of SEM image and EDS analysis; 1: magnetite, 2: wüstite.

**Figure 10:**
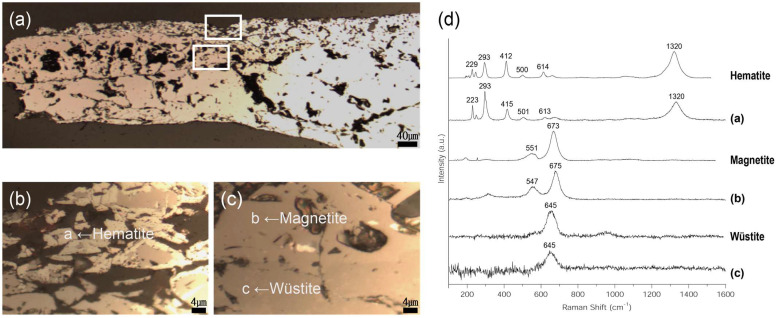
(**a**–**c**) Position of Raman micro-spectroscopy analysis of flake hammer scale (No. 3); (**a**) rectangle is position of (**b**,**c**), (**d**) Raman spectrum.

The metallographic microscope observations (Figure 9a,b) of the flake hammer scale (No. 3) revealed a distribution of a large white tetrahedral structure. The SEM-EDS analysis revealed that EDS positions 1 and 2 had similar content compositions but differed in brightness (Figure 9c, Table 4). Raman micro-spectroscopy was used to precisely identify the components’ microstructures (Figure 10), revealing differences in the brightness of all three structures that were not visible when using the metallographic microscope. Raman shifts of 223, 293, 415, 501, 613, and 1320 cm^−1^ were detected for structure 3-a, 547, 675 cm^−1^ for structure 3-b, and 645 cm^−1^ for structure 3-c, respectively, confirming that the three structures were hematite (Fe_2_O_3_), magnetite (Fe_3_O_4_), and wüstite (FeO) [22,23,24].

### 4.4. Flake Hammer Scale (No. 4)

**Figure 11:**
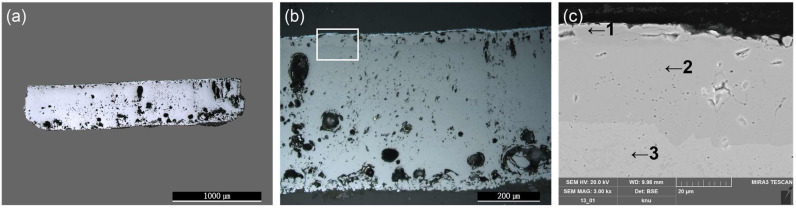
Microstructure and SEM image of flake hammer scale (No. 4): (**a**) whole image, (**b**) metallographic microscope detailed image and rectangle is position of the SEM analysisdetailed image and position of SEM analysis, (**c**) position of SEM image and EDS analysis; 1: hematite, 2: magnetite, 3: wüstite.

**Figure 12:**
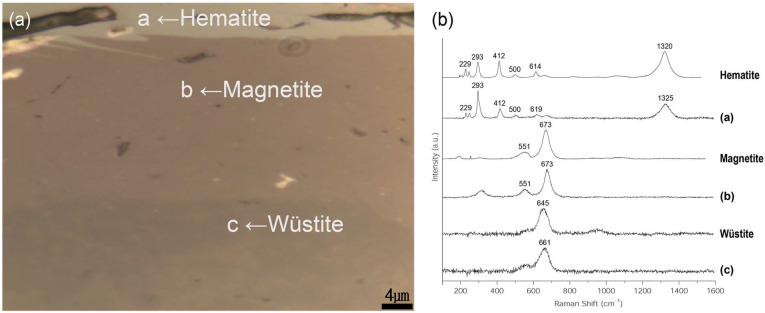
(**a**) Position of Raman micro-spectroscopy analysis of flake hammer scale (No. 4), (**b**) Raman spectrum.

The wüstite in the inner layer of granular material manifests during the first half of the forging process. During the second half of the forging process, multiple granular wüstites aggregate to form a non-crystalline structure [16]. The metallographic microscope observations (Figure 11a,b) revealed a flat, bright base structure inside, indicating that the forging process had progressed to the final stage. SEM-EDS analysis (Figure 11c, Table 5), performed on the flakes to examine their microstructure in greater detail, revealed that EDS positions 1 to 3 had similar content compositions but differed in brightness. Raman microscopy was used to precisely identify the microstructures from similar components (Figure 12). The flake hammer scales were derived from spheroidal hammer scale and were produced in smithing work. They were composed of three layers: hematite on the surface, magnetite in the middle layer, and wüstite in the inner layer [26]. Raman shift peaks of 229, 293, 412, 500, 619, and 1325 cm^−1^ were detected for structure 4-a, peaks at 551 and 673 cm^−1^ were found for structure 4-b, and a peak at 661 cm^−1^ was identified for structure 4-c, confirming that the three structures were hematite (Fe_2_O_3_), magnetite (Fe_3_O_4_), and wüstite (FeO) [22,23,24].

## 5. Discussion and Conclusions

Scientific analysis of the spheroidal hammer scale and flake hammer scale excavated from the Jincheon Songduri site was conducted to ascertain the characteristics of the smithing process.

The entire iron-making process can be divided into the two stages of smelting and smithing. However, we can divide the process into four further stages, based on the purpose of the processing: smelting, refining, forging, and shaping [2]. By-products of iron-making, such as slag, spheroidal hammer scale, and flake hammer scale, can be identified at sites where the process was carried out, allowing researchers to speculate and determine which processes were carried out at the site. When iron is heated and forged, flakes with a thickness of less than 1 mm are formed; these flakes can be divided into spheroidal hammer scale and flake hammer scale, based on their shape [21,27,28].

**Figure 13:**
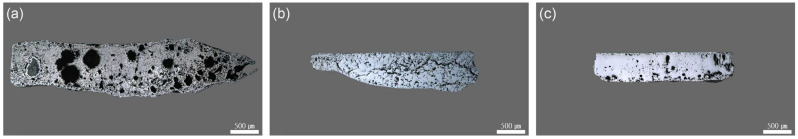
Comparison of flake hammer scale according to thickness: (**a**) flake hammer scale (No. 2), (**b**) flake hammer scale (No. 3), (**c**) flake hammer scale (No. 4).

The microscopic image of the flake hammer scale analyzed in this study is shown in Figure 13. The refining–smithing process produces a flake hammer scale with a microstructure similar to that of smelted slags, with a glassy background and fayalite and wüstite structures. Spheroidal hammer scale was frequently generated during the refining stage, but its production decreased as the process progressed [15]. The flake hammer scale sample No. 2 was approximately 1000 μm thick, and white dendritic and granular structures were visible on top of a grey fayalite background (Figure 13a). Additionally, the structure is similar to that of slag, and the spheroidal hammer scale samples excavated at the same time are a common by-product of the refining–smithing process. As a result, it is estimated that the flake hammer scale sample No. 2 was produced during the refining–smithing process. The smithing process is divided into two stages, based on the microstructure of wüstite. The first half of smithing work is considered to be a part of the initial forging process, during which wüstite exists in granular form in the inner layer. In the second half of the smithing process, repeated forging along with the shaping process results in the aggregation of the granular wüstite into a compressed, flat shape [16]. The flake hammer scale sample No. 3 was approximately 650 μm thick and was composed of a large white tetrahedral structure. Fayalite was not identified, indicating that this flake hammer scale was produced during the forging-smithing process. Moreover, it showed that the process had advanced, in comparison to flake hammer scale sample No. 2 (Figure 13b). The flake hammer scale sample No. 4 was approximately 500 μm thick, with a flat and bright base structure (Figure 13c). This flake hammer scale is believed to have been produced later in the forging–smithing process, compared to the other two flake hammer scale. Due to forging, the thickness of the flake hammer scale decreases as the process progresses through the microstructure, causing the structure to flatten.

**Figure 14:**
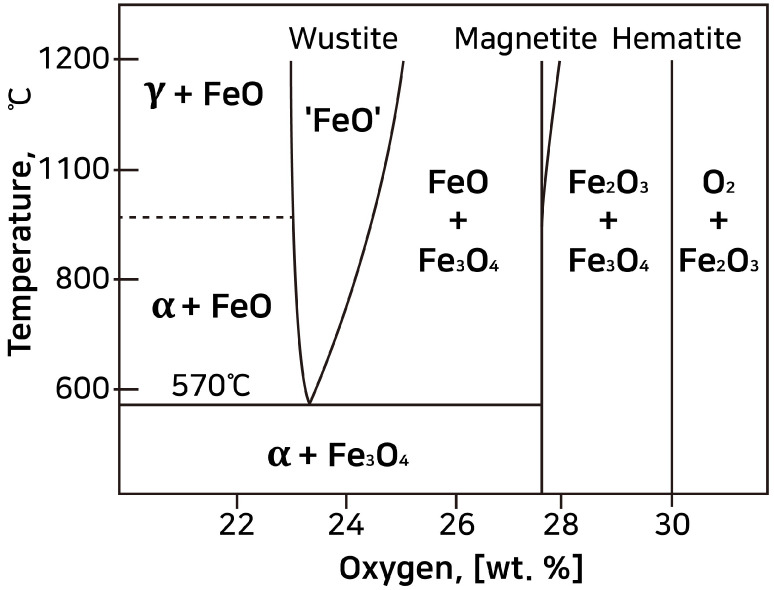
Iron–oxygen phase diagram.

Figure 14 shows the phase diagram of iron oxide according to temperature and oxygen concentration. Three types of iron oxide exist, namely, FeO, Fe_2_O_3_, and Fe_3_O_4_. Depending on the oxygen content, reduction reactions above 570 °C can induce a phase transition of hematite (Fe_2_O_3_) to magnetite (Fe_3_O_4_). Hematite and magnetite undergo a phase transition to wüstite (FeO) as the oxygen concentration decreases [21,29]. Under reduction conditions, the reaction sequence of Fe_2_O_3_ → Fe_3_O_4_ → FeO occurs, as confirmed by the structure of the flake hammer scale samples. In other words, flake hammer scale comprises three layers, with the outer, middle, and inner layer composed of hematite (Fe_2_O_3_), magnetite (Fe_3_O_4_), and wüstite (FeO), respectively [26].

**Figure 15:**
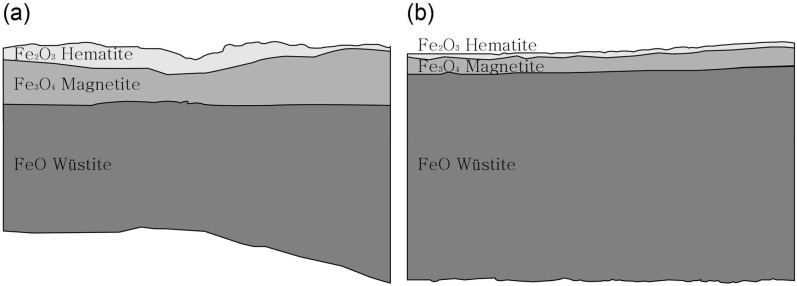
Structure of the flake hammer scale samples: (**a**) flake hammer scale (No. 3), (**b**) flake hammer scale (No. 4).

The cross-sections of the flake hammer scale samples No. 3 and No. 4 are shown in Figure 15. At the surface of flake hammer scale sample No. 2, hematite, magnetite, and wüstite were identified, with magnetite coexisting within the wüstite layer. Three layers were identified in flake hammer scale samples No. 3 and No. 4, with the outer layer, middle layer, and inner layer being identified as hematite (Fe_2_O_4_), magnetite (Fe_3_O_4_), and wüstite (FeO), respectively (Figure 15). The three flake hammer scale samples were confirmed to have been heated above 570 °C, due to the presence of hematite, magnetite, and wüstite. Smithing is a repeated heating and cooling process. When the temperature is lowered below 570 °C, the unstable wüstite must undergo a phase transition to magnetite, which requires an appropriate amount of time and temperature, as indicated by the phase diagram. However, the cooling step in the smithing process occurs quickly, preventing the wüstite from undergoing a phase transition. As a result, the wüstite layer exhibits no deformation, and all three layers are visible on the flake hammer scale sample.

This analysis of the spheroidal hammer scale and flake hammer scale excavated from an ancient iron-making site revealed that the processes involved a refining process to remove impurities, followed by a forging process that produced iron bars, requiring repeated working and confirming the division of labor involved in the smithing process. Additionally, the structural shape and thickness of the flakes provide insight into the stages of the smithing process. We anticipate that this study will be invaluable in future research on the smithing process system through additional flakes studies.

## Figures and Tables

**Table 1:** List of flake samples excavated from the Jincheon Songduri site.

No.	Find Location	Name
1	Locality 1-2, no. 2 Furnace	Spheroidal hammer scale
2	Locality 1-2, no. 2 Furnace	Flake hammer scale
3	Locality 4-1, no. 46 Residential Area	Flake hammer scale
4	Locality 2-1, no. 4 Furnace	Flake hammer scale

**Table 2:** EDS analysis results of spheroidal hammer scale (No. 1).

No.	Position	Composition (wt %)							
		Fe	O	Si	Ca	Al	K	Mg	C
1	1	39.75	46.07	2.02	1.24	0.58	0.36	-	9.98
	2	16.80	49.49	10.93	5.15	2.70	1.96	0.66	12.31

**Table 3:** EDS analysis of flake hammer scale (No. 2).

No.	Position	Composition (wt %)							
		Fe	O	Si	Ca	Al	K	Mg	C
2	1	44.11	44.11	-	-	-	-	-	11.78
	2	45.01	45.01	-	-	-	-	-	9.99
	3	20.44	51.87	11.53	7.48	-	-	0.89	7.79
	4	44.72	44.72	-	-	-	-	-	10.57

**Table 4:** EDS analysis of flake hammer scale (No. 3).

No.	Position	Composition (wt%)							
		Fe	O	Si	Ca	Al	K	Mg	C
3	1	47.11	47.11	-	-	-	-	-	5.77
	2	45.69	45.69	-	-	-	-	-	8.62

**Table 5:** EDS analysis of flake hammer scale (No. 4).

No.	Position	Composition (wt%)							
		Fe	O	Si	Ca	Al	K	Mg	C
4	1	44.23	44.23	-	-	-	-	-	11.54
	2	44.96	44.96	-	-	-	-	-	10.08
	3	43.45	43.45	-	-	-	-	-	13.10

## Data Availability

Not applicable.
